# Mesenteric panniculitis: Radiological and clinical evaluation of 15 patients: A case series

**DOI:** 10.1097/MD.0000000000045271

**Published:** 2025-10-24

**Authors:** Ramazan Saygin Kerimoğlu, Süleyman Kargin, Yusuf Tanrikulu, Abdullah Gürhan Duyan, Gökhan Yilmaz

**Affiliations:** a Department of Gastrointestinal Surgery, Health Sciences University, Konya City Hospital, Konya, Turkey; b Department of General Surgery, Health Sciences University, Konya City Hospital, Konya, Turkey; c Department of General Surgery, Faculty of Medicine, Baskent University, Konya, Turkey; d Department of General Surgery, Faculty of Medicine, Medipol University, Istanbul, Turkey.

**Keywords:** abdominal pain, acute abdomen, mesenteric fibrosis, mesenteric lipodystrophy, mesenteric panniculitis

## Abstract

The goal of our study was to compare the clinical findings, laboratory, and imaging results of 15 individuals with uncommon mesenteric panniculitis (MP) to the literature. A case series. The General Surgery Department of Konya City Hospital, Turkey, between January 2014 and December 2021. Patients who were followed up on and treated for MP in a tertiary university hospital’s general surgery clinic. A total of 2361 patient computer tomography scans were evaluated, and archive records were used to compile data for 15 patients with MP. The patients’ demographics, complaints, hospitalization requirements, therapy methods, laboratory, and radiographic characteristics were all analyzed. Abdominal pain was the most common complaint among the patients (80%). This was followed by nausea, vomiting (40%), chest pain (33%), and diarrhea (7%). Fat halo sign was seen in 8 patients due to mesenteric vascular involvement. In 5 patients, an image consistent with mesenteric adenitis >10 mm was found. Four patients exhibited a pseudo capsule appearance, and 2 had a mesenteric mass pushing on the intestinal loops. MP is a disease that should be considered in the differential diagnosis of patients who present to the emergency department with abdominal pain, as it will ensure that the patient receives the appropriate treatment and reduce the high rate of patients discharged with the diagnosis of nonspecific abdominal pain.

## 1. Introduction

Mesenteric panniculitis (MP) is a fibrotic, nonneoplastic inflammatory disease. Depending on the degree of inflammation and fibrosis, various terminology has been used, such as mesenteric lipodystrophy (predominantly fat necrosis), sclerosing mesenteritis, or mesenteric fibrosis. The examination of histology is rarely documented in the literature.^[1]^

It can induce gastrointestinal and systemic symptoms such as abdominal discomfort, nausea, vomiting, diarrhea, weight loss, and fever.^[2]^ It has been reported that 20%–25% of patients require hospitalization after admission, while no pathology is discovered in 35%–40% of them despite the tests performed; the pain usually resolves spontaneously and is classified as nonspecific abdominal pain.^[3,4]^

The prevalence of sclerosing mesenteritis was reported to be 1% in an autopsy series.^[5]^ However, since most of individuals are asymptomatic, it is still classified as an uncommon condition whose etiology is not completely comprehended.

MP is a benign condition with no definite nomenclature or classification. This disease, which is characterized by the coexistence of fat necrosis, chronic inflammation, and fibrosis in the mesenteric tissue, can be characterized by either of these 3 components depending on the stage of the disease, and the disease’s designation varies accordingly.^[6]^ It has been observed that if the inflammation of the fatty tissue of the mesentery and necrosis predominates, the condition is known as MP, however, if fibrosis predominates, the disease is known as “retractile mesenteritis.”^[7]^ Although the cause of MP is unknown, it is linked to a number of disorders. Underlying or associated illnesses include vasculitis, granulomatous diseases, rheumatological diseases, malignancies, pancreatitis, past abdominal surgery or trauma, ischemia damage, and infections.^[8]^

The goal of our study was to compare the clinical findings, laboratory, and imaging results of 15 individuals with uncommon MP to the literature. We observe that, because this is a rare condition, this issue is being clarified by generating compilations of case reports in the literature, and we believe that providing the data we have can at least contribute to these studies.

## 2. Methods

### 2.1. Study design

Patients who were followed up on and treated for MP in a tertiary university hospital’s general surgery clinic between January 1, 2014 and December 31, 2021, were included in the retrospective analysis. Local ethics committee approval number 2025/024 was received. A total of 2361 patient computer tomography (CT) scans were evaluated, and archive records were used to compile data for 15 patients who were tomographically compatible with MP. The study excluded patients under the age of 18, those with secondary peritonitis, those with known intra-abdominal cancer, and those with mesenteric lymphadenitis alone.

The patients’ demographics, complaints, hospitalization requirements, therapy methods, laboratory, and radiographic characteristics were all analyzed.

### 2.2. Statistical analysis

Statistical Package for Social Science for Windows 15.0 was used to analyze the data. The chi-square test (and/or Fisher exact test) was used to analyze categorical variables, which were reported as numbers (n) and percentages (%). The means of 2 independent groups were compared using the Student *t* test, and numerical variables were reported as mean ± standard deviation.

### 2.3. Diagnostic criteria

The best diagnostic approach is abdominal CT, and CT findings are diagnostic. The role of biopsy in MP patients is debatable. As a result, no biopsy or surgery was required for diagnosis. The CT criteria for the diagnosis of MP were determined as follows and the diagnosis was made on the following criteria:

1. Increased density and heterogeneity in the root periphery of the small intestine mesentery, with greater attenuation in fat planes than retroperitoneal fat.
2. The superior mesenteric vessels to be wrapped without vascular involvement (fat halo sign).
3. Mass displacing bowel loops without evidence of invasion.
4. Lymph nodes with a short axis of 10 mm.
5. Pseudo capsule (the area observed between the normal mesentery and the inflamed area in the peripheral region).^[9]^

## 3. Results

Our study included 15 patients with MP. Table 1 presents the demographic information for the patients. According to these findings, 6 (40%) of the patients were male, 9 (60%) were female, and the average age was 66.40 ± 13.20 years. Abdominal pain was the most common complaint among the patients (80%). This was followed by nausea, vomiting (40%), chest pain (33%), and diarrhea (7%). Three patients had systemic symptoms such as fatigue and fever. (20%). One patient (7%) was observed to be asymptomatic. The majority of the patients had no comorbid diseases. When the patients’ previous procedures were investigated, it was discovered that 4 patients (26%) had a history of appendectomy and 2 patients (13%) had a history of cholecystectomy. There were no patients who had undergone major abdominal surgery as a result of malignancy. Seven patients (47%) were hospitalized, while the remaining patients were treated as outpatients with oral medications. Surgery was undertaken on just 2 patients since there was no decrease in inflammatory parameters throughout clinical follow-up and because the clinic deteriorated. Two patients who had surgery required no intestinal resection and solely mesenteric abscess drainage was performed.

**Table 1:** Demographic characteristics of patients.

Gender (male/female)	6 (40%)/9 (60%)
Mean age (yr)	66.40 ± 13.20
Complaints	
Abdominal pain	12 (80%)
Chest pain	5 (33%)
Nausea–vomiting	6 (40%)
Diarrhea	1 (7%)
Fever	3 (20%)
Weakness	3 (20 %)
Asymptomatic	1 (7%)
Comorbid disease	
Diabetes mellitus	2 (13%)
Hypertension	2 (13%)
None	13 (74%)
Previous surgery	
Appedectomy	4 (26%)
Cholecystectomy	2 (13%)
Hospitalization	7 (47%)
Surgical treatment	2 (13%)
Length of stay (d)	1.67 ± 1.76

Table 2 displays the patients’ laboratory results. According to these findings, the patients’ mean liver function tests and renal function tests were within normal ranges. C-reactive protein (CRP) and white blood cell levels were elevated in all cases.

**Table 2:** Laboratory results of patients.

WBC (×1000/L)	11.85 ± 4.57
CRP (mg/L)	56.09 ± 14.68
NLR	5.34 ± 5.16
MPV (fL)	8.60 ± 1.71
Hg (g/dL)	14.15 ± 1.35
Urea (mg/dL)	37.07 ± 8.40
Creatinin (mg/dL)	0.94 ± 0.18
Glucose (mg/dL)	131.67 ± 27.93
ALT (U/L)	25.27 ± 15.61
AST (U/L)	23.93 ± 11.68

ALT = alanine aminotransferase, AST = aspartate aminotransferase, CRP = C-reactive protein, Hg = hemoglobin, MPV = mean platelet volume, NLR = neutrophil lymphocyte ratio, WBC = white blood cell.

Table 3 presents the sagittal and coronal tomography portions of each patient’s radiological data.

**Table 3:**
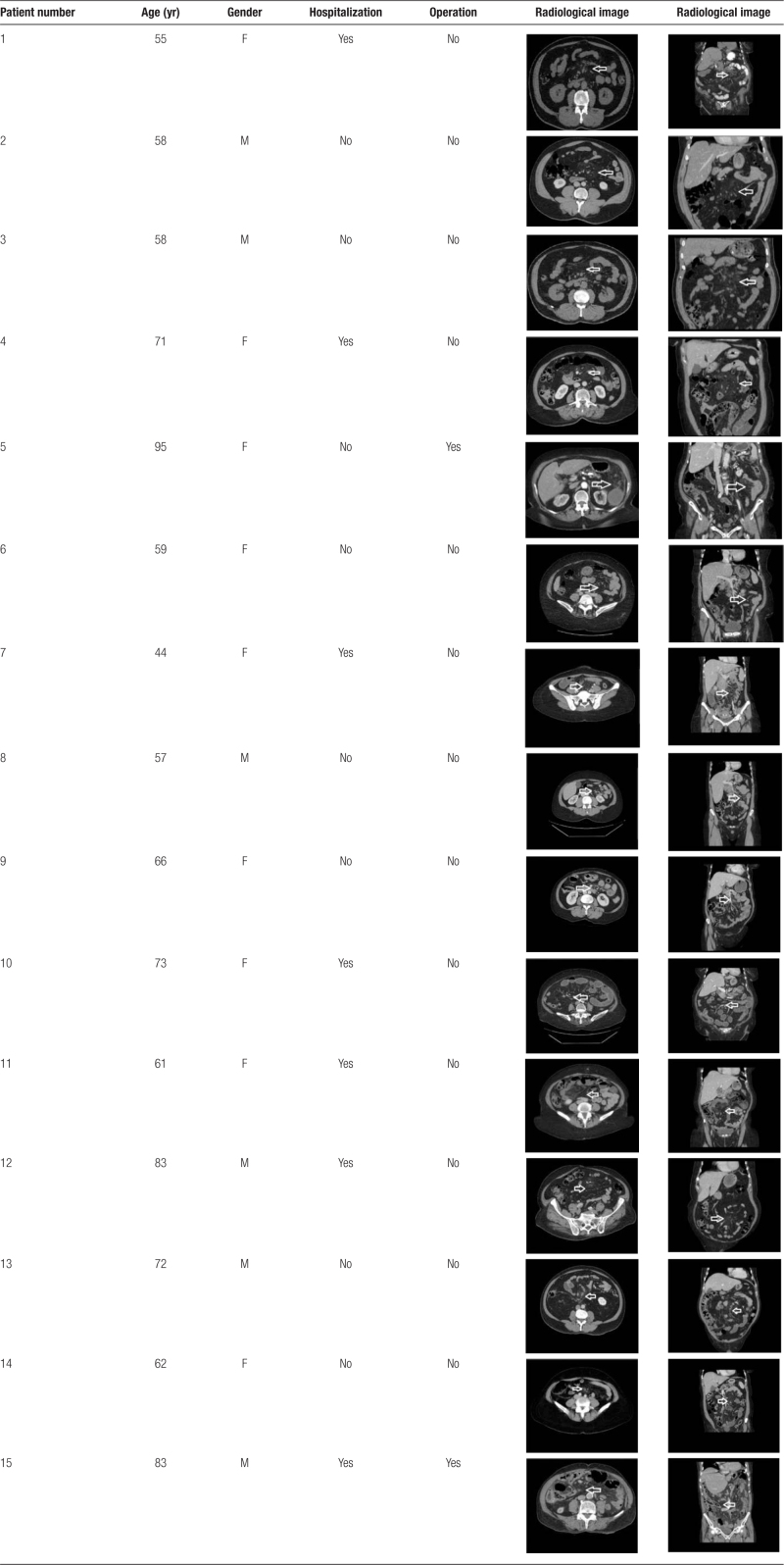
Data of all patients.

The mesenteric root fat planes were denser and more heterogeneous in all patients, with higher attenuation than retroperitoneal fat. Fat halo sign was seen in 8 patients due to mesenteric vascular involvement. In 5 patients, an image consistent with mesenteric adenitis >10 mm was found. Four patients exhibited a pseudo capsule appearance, and 2 had a mesenteric mass pushing on the intestinal loops.

## 4. Discussion

Since MP is a rare condition, it is frequently attempted to be clarified by compiling case reports from the literature. We believed that sharing the data and experience we have with this patient group, for whom we handle the diagnosis and treatment process, would at the very least help this research. MP is a disease that should be investigated in the differential diagnosis of patients with abdominal pain who present to the emergency service. Furthermore, we believe that using nonsteroidal anti-inflammatory drugs (NSAIDs), antibiotics, and corticosteroids as the primary treatment may be sufficient in many patients.

Sclerosing mesenteritis is most frequent between the ages of 50 and 70, with a mean age of 65.^[10,11]^ The mean age in our study was 66.40. In terms of sex, Durst et al discovered a male/female ratio of 1.8 in their research scanning the literature.^[12]^ Daskalogiannaki et al found that MP was 65.3 % more prevalent in women in their study of 49 patients.^[6]^ Six (40%) of the 15 patients in our study were male, whereas 9 (60%) were female. Although most studies have revealed that males had a higher prevalence of MP, there is no uniformity in the literature.^[7,10,11]^

Abdominal pain is the most prevalent presenting symptom in these patients, accounting for 30% to 70% of all cases.^[7,11,12]^ Eighty percent of our participants in our study complained of abdominal pain. In 6 patients, this was followed by nausea and vomiting (40%). Only 1 patient (7%) had diarrhea but no constipation. Systemic symptoms such as fever and fatigue were experienced by 3 (20%) of the patients. When Akram et al^[11]^ examined the symptoms of 92 patients, they discovered that 70% had abdominal pain, 26% had bloating and distention complaints, 25% had diarrhea, and 23% had weight loss. They indicated that they observed no symptoms in 9 (10%) of the patients. According to our study and the literature, just 1 patient (7%) was asymptomatic in our study.

Laboratory tests are frequently in a normal range and do not contribute to diagnosis.^[8,13]^ Patients suffering from an inflammatory condition may have elevated levels of CRP and erythrocyte sedimentation rate.^[14,15]^ In 1 study, erythrocyte sedimentation rate was found to be elevated in more than 60% of instances of MP. Anemia and high leukocyte counts have also been documented in the literature.^[8,11,15]^ The laboratory results of the MP patients included in our study are presented in Table 2. Despite the presence of elevated CRP (56.09 ± 14.68) and leukocytosis (11.85 ± 4.57), we believe that the significance of laboratory parameters to the diagnosis of MP is limited because these tests are imprecise.

Imaging tests are the most useful noninvasive diagnostic procedures. Although plain abdomen X-rays or barium X-rays are not diagnostic, screening with ultrasonography and CT is incredibly useful in diagnosis.^[16]^ According to several studies, CT alone can be diagnostic because its findings are extremely specific.^[6,13]^ Among the CT findings specific to MP are a well-circumscribed fatty mass surrounding the superior mesenteric vessels, displacement of adjacent intestinal loops, well-circumscribed soft tissue nodules smaller than 5 mm, and the appearance of a very thin strip surrounding the muscle, known as the hypodense “fatty halo” and “pseudo tumoral strip” surrounding the nodule or vessels. These pathognomonic CT features were used to make the diagnosis in our patients (Table 3).

In patients with symptomatic sclerosing mesenteritis, Danford et al^[2]^ employed glucocorticoids (40 mg prednisone per day) and tamoxifen (10 mg twice a day) as the first pharmacological treatment. For pain management, all patients in our research received NSAIDs and antibiotherapy. While cephalosporin antibiotics were given to 12 patients, quinolone antibiotics were recommended because 2 patients had a history of penicillin allergy. Except for 1 out of 8 outpatients, the symptoms were completely reversed. This patient was given 40 mg of Prednisolone orally daily for 1 month, and the patient’s pain complaint faded by the end of the first month. Because of the intensity of their complaints and high inflammation measures, 7 patients were hospitalized and followed up on. NSAIDs and antibiotherapy were the primary treatments. Due to the incapacity of 2 inpatients to regress in inflammatory parameters and the deterioration of the clinic, surgical intervention was scheduled. Two patients who had surgery required no intestinal resection and solely mesenteric abscess drainage was performed. One inpatient was started on oral prednisolone 40 mg/d after complaining of continuous abdominal pain. Tamoxifen was prescribed when the patient’s complaints of abdominal pain persisted after the first month. At the third month follow-up, the patient’s symptoms had entirely resolved. Akram et al^[11]^ found that deep venous thromboembolism developed in 3 (16%) of 19 tamoxifen-treated patients, and pulmonary thromboembolism in 2 individuals, despite the absence of any risk factors. One patient had leukopenia as a result of azathioprine. Treatment-related mortality was observed in 2 patients. They reported that 1 patient died in the first month from thrombotic thrombocytopenic purpura caused by tamoxifen medication, while the other died in the fifth month from sepsis caused by the usage of thalidomide and azathioprine. Only 1 patient required the usage of tamoxifen because his/her pain could not be controlled with NSAIDs and Prednisolone treatment. Except for dyspeptic complaints and mood changes, we found no drug-related morbidity. To stabilize the condition, a variety of immunosuppressive medications are employed. However, there is limited case series data supporting their usage in the treatment of MP symptoms, and these medications have not been directly compared. In patients who cannot tolerate tamoxifen due to side effects or who have tamoxifen contraindications, alternative treatment techniques may be required. Although numerous case studies in the literature have revealed that chemotherapeutics such as azathioprine^[17]^ progesterone,^[18]^ cyclophosphamide,^[19]^ infliximab,^[20]^ and pentoxifylline^[21]^ are effective in the treatment, their efficacy is limited. Only in patients who have failed corticosteroid and tamoxifen treatment do we recommend these medications. However, we state that these agents are not required in our case series.

Previous abdominal surgery or abdominal trauma was described in nearly 30% of individuals with MP in the review by Sharma et al^[10]^ In a study of 92 patients with sclerosing mesenteritis, 38 (41%) had abdominal surgery, which included cholecystectomy, appendectomy, hysterectomy, and colectomy.^[11]^ We also discovered that 4 of the 15 patients we examined underwent appendectomy and 2 had a cholecystectomy (40%). Among our cases, however, there was no patient who had undergone cancer surgery. There are studies in the literature that show no correlation between previous abdominal surgery or trauma and MP.^[22]^ On the other hand, other studies have found malignancy at widely disparate rates.^[6,10]^ The most common associated malignancy is non-Hodgkin lymphoma, but it has been linked to a variety of cancers, including breast cancer, melanoma, lung adenocarcinoma, renal carcinoma, multiple myeloma, hepatocellular carcinoma, prostate adenocarcinoma, ovarian carcinoma, endometrial carcinoma, cervical carcinoma, and gastrointestinal adenocarcinomas.^[23]^ In our case series, however, no cancer or comorbid disease was found in any of the patients. Two patients with type 2 diabetes mellitus were being monitored and treated (13%). According to Buchwald et al,^[24]^ MP is not a paraneoplastic indication and is most likely an epiphenomenon seen in CT scans used for cancer staging.^[25]^

## 5. Conclusion

MP is a disease that should be considered in the differential diagnosis of patients who present to the emergency department with abdominal pain, as it will ensure that the patient receives the appropriate treatment and reduce the high rate of patients discharged with the diagnosis of nonspecific abdominal pain. We wanted to draw attention to this issue because it is easy to overlook due to the nonspecificity of the findings and the mild nature of the symptoms. Since MP is an uncommon condition, more extensive investigations are required, and we believe that sharing data on this issue, albeit restricted, will contribute to future studies.

## Author contributions

**Conceptualization:** Ramazan Saygin Kerimoğlu.

**Data curation:** Süleyman Kargin.

**Formal analysis:** Ramazan Saygin Kerimoğlu.

**Funding acquisition:** Ramazan Saygin Kerimoğlu.

**Investigation:** Ramazan Saygin Kerimoğlu.

**Methodology:** Ramazan Saygin Kerimoğlu.

**Project administration:** Yusuf Tanrikulu.

**Resources:** Abdullah Gürhan Duyan.

**Software:** Gökhan Yilmaz.

**Validation:** Gökhan Yilmaz.

**Visualization:** Süleyman Kargin.

**Writing – original draft:** Ramazan Saygin Kerimoğlu.

**Writing – review & editing:** Yusuf Tanrikulu.
